# Bonding to Demineralized Dentin: Impact of Immediate and Delayed Dentin Sealing over Time

**DOI:** 10.3390/dj13080354

**Published:** 2025-08-05

**Authors:** Erika Pérez-Soto, Rim Bourgi, Louis Hardan, Carlos Enrique Cuevas-Suarez, Ana Josefina Monjáras-Ávila, Miguel Ángel Fernández-Barrera, Nicolas Nassar, Monika Lukomska-Szymanska, Rima Daoui, Naji Kharouf, Youssef Haikel

**Affiliations:** 1 Dental Materials Laboratory, Academic Area of Dentistry, Autonomous University of the State of Hidalgo State, San Agustín Tlaxiaca 42160, Mexico; erika9719@outlook.com (E.P.-S.); cecuevas@uaeh.edu.mx (C.E.C.-S.); ana_monjaras@uaeh.edu.mx (A.J.M.-Á.); miguel_fernandez10334@uaeh.edu.mx (M.Á.F.-B.); 2 Department of Restorative and Esthetic Dentistry, Faculty of Dental Medicine, Saint-Joseph University of Beirut, Beirut 1107 2180, Lebanon; louis.hardan@usj.edu.lb; 3 Department of Restorative Sciences, Faculty of Dentistry, Beirut Arab University, Beirut 115020, Lebanon; 4 Department of Biomaterials and Bioengineering, INSERM UMR_S 1121, University of Strasbourg, 67000 Strasbourg, France; dentistenajikharouf@gmail.com; 5 Department of Digital Dentistry, AI, and Evolving Technologies, Faculty of Dental Medicine, Saint-Joseph University of Beirut, Beirut 1107 2180, Lebanon; nicolas.nassar.dds@hotmail.com; 6 Department of Orthodontics, Faculty of Dental Medicine, Saint-Joseph University of Beirut, Beirut 1107 2180, Lebanon; 7 Craniofacial Research Laboratory, Faculty of Dental Medicine, Saint-Joseph University of Beirut, Beirut 1107 2180, Lebanon; 8 Department of General Dentistry, Medical University of Lodz, 92-213 Lodz, Poland; monika.lukomska-szymanska@umed.lodz.pl; 9 Independent Researcher, Beirut 1103, Lebanon; rimadawi3@gmail.com; 10 Department of Endodontics and Conservative Dentistry, Faculty of Dental Medicine, University of Stras-bourg, 67000 Strasbourg, France; 11 Pôle de Médecine et Chirurgie Bucco-Dentaire, Hôpital Civil, Hôpitaux Universitaire de Strasbourg, 67000 Strasbourg, France

**Keywords:** indirect restorations, immediate dentin sealing, demineralized dentin

## Abstract

**Background/Objectives:** Immediate dentin sealing (IDS) has been widely investigated in sound dentin; however, its efficacy on demineralized dentin remains insufficiently explored. This in vitro experimental study aimed to evaluate the shear bond strength (SBS) of indirect composite resin restorations bonded to demineralized dentin using IDS, assessed at 24 h and after 6 months of aging. **Methods:** Twenty-five extracted premolars were randomly divided into five groups: (1) control (no sealing), (2) IDS applied to sound dentin (sound-IDS), (3) IDS applied to demineralized dentin (carious-IDS), (4) delayed dentin sealing (DDS) on sound dentin (sound-DDS), and (5) DDS on demineralized dentin (carious-DDS). SBS values were analyzed using a three-way analysis of variance (ANOVA) with dentin condition (sound vs. demineralized), aging time (24 h vs. 6 months), and sealing strategy (control, IDS, DDS) as independent variables. Statistical analyses were performed using SigmaPlot 12.0, with significance set at *p* < 0.05. **Results:** The results showed that IDS led to significantly higher SBS than DDS (*p* < 0.05). Bond strength was significantly influenced by dentin condition (*p* < 0.05), and all interactions between variables—particularly between dentin condition and sealing strategy, and between aging time and treatment—were statistically significant (*p* < 0.001). Overall, bond strength was higher at 24 h than after 6 months. IDS showed optimal performance in sound dentin, while DDS resulted in better long-term outcomes in demineralized dentin. **Conclusions:** These findings suggest that DDS may be the more effective approach in cases of carious or demineralized dentin.

## 1. Introduction

Indirect restorations represent a fundamental component of modern restorative dentistry, particularly for the management of teeth with extensive structural damage due to caries, trauma, or wear. These restorations are fabricated outside the oral cavity, using a working model obtained through conventional impressions or digital scanning, and are subsequently bonded to the prepared tooth using adhesive luting agents [1,2]. The advantages of indirect restorations include superior control of occlusal anatomy, improved proximal contact adaptation, and enhanced fracture resistance—especially in posterior teeth exposed to high masticatory loads [3]. When properly bonded, these restorations offer excellent longevity and esthetic outcomes.

In current minimally invasive dentistry, the traditional concept of extensive caries removal—originally proposed by G.V. Black—has been replaced by selective caries removal strategies that aim to preserve healthy dentin and enamel [4]. Selective removal techniques focus on eliminating infected dentin while retaining affected but remineralizable tissue, especially near the pulp, thereby reducing the risk of pulp exposure and postoperative sensitivity [5]. This approach is particularly relevant in deep lesions, where complete debridement may compromise the biomechanical integrity of the tooth or endanger pulpal health.

To optimize adhesion in indirect procedures, the Immediate Dentin Sealing (IDS) technique was introduced in the 1990s. This protocol involves applying an adhesive system to freshly cut dentin immediately after tooth preparation and before impression taking or digital scanning [6]. IDS has been associated with several benefits, including improved bond strength, reduced dentin sensitivity, enhanced marginal adaptation, and protection of the dentin-pulp complex during the provisional phase [7,8]. Moreover, IDS may reduce the risk of contamination from temporary cements and allow for stress-free polymerization of the adhesive layer prior to cementation.

Despite the proven advantages of IDS in sound dentin, there is a lack of consistent data regarding its performance in demineralized dentin substrates.

Demineralized dentin is characterized by a reduction in mineral content, exposing a porous collagen matrix that is often incompletely infiltrated by adhesive resins. This collagen network is fragile and prone to enzymatic degradation, while increased permeability and altered water content hinder proper adhesive penetration and polymerization. These structural and chemical changes reduce the bonding substrate’s mechanical integrity and can impair the durability of adhesive interfaces [9].

This is clinically relevant, as selective caries removal frequently results in bonding to partially demineralized tissue. The compromised collagen network, altered permeability, and lower mineral content of carious dentin may reduce the effectiveness of adhesive infiltration and polymerization, potentially affecting the long-term performance of the adhesive interface. Selective caries removal has become a widely accepted conservative treatment approach aimed at preserving tooth structure and avoiding pulp exposure. By intentionally leaving some demineralized dentin on the pulp, this technique reduces the risk of pulpitis and promotes reparative dentin formation. However, bonding to this partially demineralized tissue poses challenges because the compromised collagen network, altered permeability, and reduced mineral content can impair adhesive infiltration and polymerization. Consequently, the adhesive interface may be more susceptible to degradation, leading to microleakage, secondary caries, and ultimately restoration failure over time. Understanding how bonding protocols like IDS perform on these substrates is therefore essential to improve restoration longevity and clinical outcomes in minimally invasive dentistry [10,11,12,13,14]. Therefore, more evidence is needed to determine whether IDS remains effective under these altered conditions.

Therefore, the aim of this in vitro study was to evaluate the shear bond strength (SBS) of indirect composite resin restorations bonded to demineralized dentin using the IDS technique, with a comparison to delayed dentin sealing (DDS) after aging. The null hypothesis was that there would be no statistically significant differences in SBS among the different dentin conditions (sound vs. demineralized), sealing protocols (IDS vs. DDS), or aging times (24 h vs. 6 months).

## 2. Materials and Methods

This in vitro study evaluated the SBS of indirect composite resin restorations to dentin, comparing IDS and DDS techniques in both sound and demineralized dentin, with and without aging. The study protocol involved tooth selection and preparation; the induction of artificial carious dentin; the application of sealing techniques; the fabrication of indirect restorations; cementation procedures; aging; and mechanical testing. The restoration materials used in this study are described in Table 1.

### 2.1. Sample Selection and Experimental Groups

Twenty-five non-carious human premolars extracted for orthodontic purposes were selected, cleaned, and stored in 0.1% thymol solution at 4 °C until use. Teeth were randomly assigned to five experimental groups (n = 5 per group): control (no sealing), IDS on sound dentin (sound-IDS), IDS on demineralized dentin (carious-IDS), DDS on sound dentin (sound-DDS), and DDS on demineralized dentin (carious-DDS).

Randomization was performed using a block randomization method. In the sound dentin groups, the teeth were stored in deionized water, which was refreshed every 8 h, repeated over 14 days. In the carious dentin groups, an artificial caries protocol was applied.

### 2.2. Preparation of Artificial Caries-Affected Dentin

Artificial demineralized dentin was created using a pH-cycling method adapted from Lenzi et al. [15]. This protocol involves cyclic exposure to demineralizing and remineralizing solutions to simulate the dynamic process of caries formation. The occlusal enamel was removed using a precision cutter (IsoMet 1000, Buehler, Lake Bluff, IL, USA) under water cooling to expose a flat dentin surface. The pulp chambers were sealed with composite resin to stabilize the structure. The exposed dentin was polished with 800- and 1200-grit silicon carbide papers for 15 s each.

All surfaces except the occlusal dentin were covered with acid-resistant varnish. The specimens were immersed in a demineralizing solution (2.2 mM CaCl_2_, 2.2 mM NaH_2_PO_4_, 50 mM acetic acid, and pH 4.5) for 8 h, followed by immersion in a remineralizing solution (1.5 mM CaCl_2_, 0.9 mM NaH_2_PO_4_, 0.15 M KCl, and pH 7.0) for 16 h. This cycle was repeated daily for 14 days, and all solutions were renewed every 24 h. After cycling, the specimens were embedded in epoxy resin using PVC molds, leaving the bonding surface exposed.

### 2.3. Immediate Dentin Sealing (IDS) and Delayed Dentin Sealing (DDS)

In the IDS groups, a thin layer of Single Bond Universal adhesive (3M ESPE, St. Paul, MN, USA) was applied to the dentin surface with a microbrush for 15 s, gently air-dried, and light-cured for 10 s (Bluephase Style, Ivoclar Vivadent; 1000 mW/cm^2^). Glycerin gel was applied, and an additional light curing of 40 s was performed to ensure complete polymerization.

In the DDS and control groups, no adhesive was applied at this stage. For all groups, a provisional restoration (Systemp Onlay, Ivoclar Vivadent) was fabricated and light-cured on the flat dentin surface for 20 s. Specimens were stored in distilled water at 37 °C for 3 weeks to simulate the temporary phase.

### 2.4. Fabrication of Indirect Restorations and Cementation

Resin composite cylinders (1.5 mm diameter × 1 mm height) were fabricated using Filtek Z250 XT (3M ESPE) placed into a silicone mold. The resin was gently compacted with a stainless-steel spatula, covered with a Mylar strip and a glass slide to obtain a flat surface, and light-cured for 20 s. After removal from the mold, the cylinders were air-abraded for 20 s with 50 µm aluminum oxide particles at 60 PSI from a distance of 1 cm, then separated into experimental groups.

After the temporary phase, provisional restorations were removed with a scaler, and the dentin surfaces were cleaned using pumice paste. In the IDS groups, a new layer of Single Bond Universal adhesive was applied for 15 s and gently air-dried for 10 s but was not light-cured, following the manufacturer’s protocol for dual-cure resin cements.

**Figure 1:**
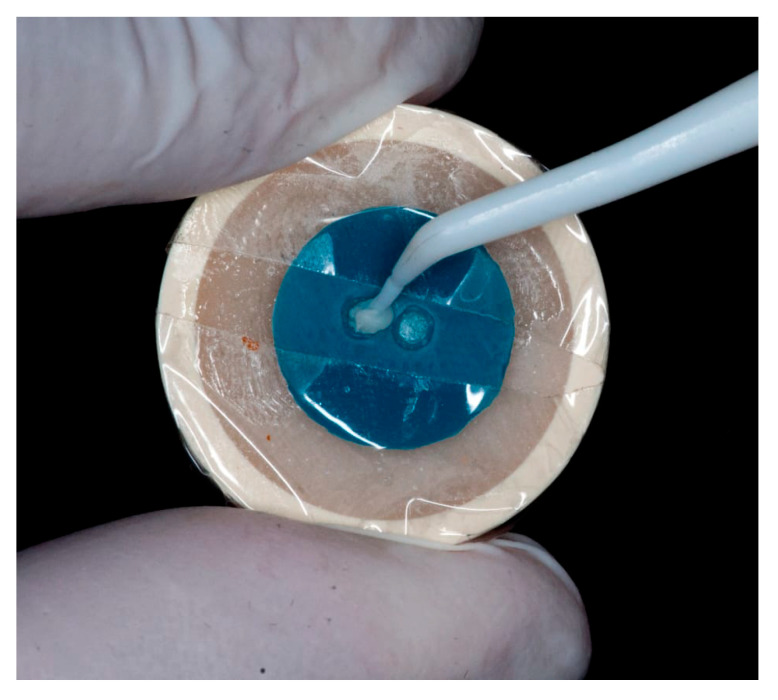
Prefabricated silicone positioning guide to ensure standardized alignment and reproducibility in bonding process.

Cementation was performed using Theracem (Bisco, Schaumburg, IL, USA), a dual-cure self-adhesive resin cement. A thin layer of cement was placed inside the resin cylinder, which was then seated onto the dentin surface using a prefabricated silicone positioning guide to ensure standardized alignment and reproducibility (Figure 1).

All bonding and restorative procedures were performed by the same calibrated operator to ensure standardization and reduce operator-related bias.

To simulate clinical seating pressure, a standardized load of 500 g was applied vertically over each cylinder for 20 s using a calibrated weight before initial light-curing. A tack-cure of 2 s was performed to facilitate the removal of excess cement, followed by final polymerization with a curing light for 20 s.

### 2.5. Shear Bond Strength Testing

After cementation, all specimens were stored in distilled water at 37 °C. Half of the specimens (n = 5 per group) were tested after 24 h, and the remaining half after 6 months of storage to simulate long-term aging. During the storage period, water was replaced weekly to avoid contamination or microbial growth.

Before testing, specimens were removed from the storage medium, gently dried, and inspected under magnification to verify the integrity of the bonded interface. The shear SBS test was performed using a universal testing machine (Instron 4465, Norwood, MA, USA) equipped with a 1 kN load cell.

To apply the load, a 0.2 mm diameter orthodontic stainless-steel wire was positioned around the base of each resin cylinder, flush against the adhesive interface. The wire was anchored to a custom jig designed to maintain a consistent load application angle perpendicular to the bonding surface. The force was applied at a crosshead speed of 1 mm/min until failure occurred.

**Figure 2:**
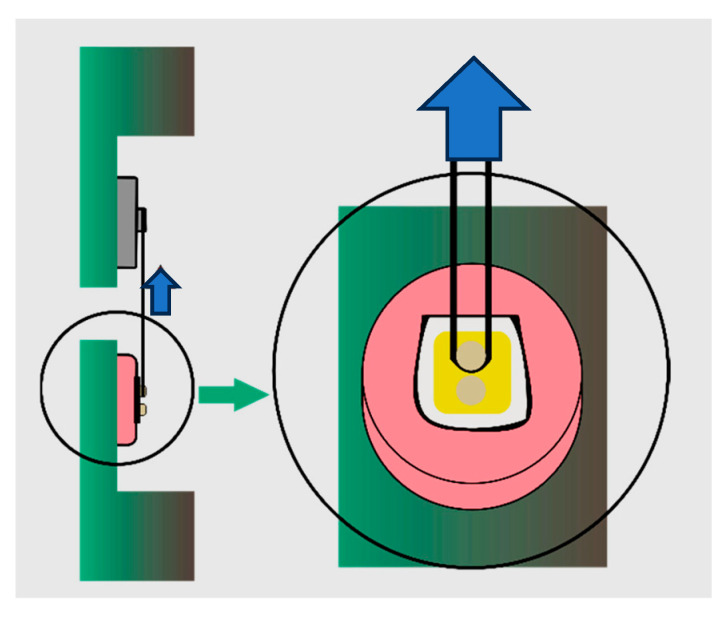
Shear bond strength test. Blue arrows indicate the direction of the forces.

The maximum load at failure was recorded in Newtons (N) and converted to megapascal (MPa) by dividing the force by the bonded surface area (1.77 mm^2^). All tests were performed under controlled environmental conditions (temperature 23 ± 1 °C, relative humidity 50 ± 5%). To minimize bias, the specimens were coded and randomized prior to testing, and the investigators conducting the shear bond strength (SBS) tests were blinded to the group allocations throughout the procedure (Figure 2).

The failure mode of each specimen was examined under a stereomicroscope at 40× magnification and classified as: adhesive failure (at the dentin–resin interface), cohesive failure (within the resin or dentin), or mixed failure (a combination of adhesive and cohesive) [16].

### 2.6. Statistical Analysis

All statistical analyses were performed using SigmaPlot 12.0 (Systat Software, San Jose, CA, USA). Data on SBS were first tested for normality using the Shapiro–Wilk test and for homogeneity of variances using Levene’s test to confirm the assumptions for parametric analysis.

A three-way analysis of variance (ANOVA) was conducted to evaluate the influence of three independent factors: Dentin condition (sound vs. demineralized), Sealing strategy (control, IDS, DDS), Aging time (24 h vs. 6 months), as well as their interaction effects on SBS values. When significant differences were detected (*p* < 0.05), post hoc pairwise comparisons were performed using the Holm–Sidak method to identify specific group differences.

Due to the presence of significant interaction effects between variables, additional two-way ANOVA analyses were carried out separately for the 24 h and 6-month groups to clarify the behavior of the sealing strategies under each aging condition. Quantitative results were expressed as mean ± standard deviation (SD). The level of statistical significance was set at *p* < 0.05 for all comparisons.

## 3. Results

The SBS values were analyzed to evaluate the effects of dentin condition (sound vs. demineralized), sealing strategy (control, IDS, DDS), and aging time (24 h vs. 6 months). The three-way ANOVA revealed that all three main factors had a statistically significant impact on SBS (*p* < 0.001). Furthermore, significant interactions were observed between dentin condition and sealing strategy (*p* < 0.001), as well as aging time and sealing strategy (*p* < 0.001). The interaction between all three factors was not statistically significant (*p* = 0.341) (Table 2).

Given the presence of interaction effects, further two-way ANOVA analyses were conducted separately for each aging condition to clarify how dentin condition and sealing technique influenced SBS at 24 h and after 6 months.

At 24 h, both dentin condition (*p* = 0.009) and sealing strategy (*p* < 0.001) were statistically significant. The highest SBS values were observed in the sound-IDS group (13.69 ± 1.48 MPa) and the sound-control group (13.85 ± 2.35 MPa), with no statistically significant difference between them. The 95% confidence intervals (CIs) for these groups were approximately 11.62 to 16.08 MPa and 12.14 to 15.24 MPa, respectively, indicating precise and clinically robust bonding performance (Cohen’s d = 0.07, negligible effect size).

In contrast, the lowest SBS values were recorded in the DDS groups, regardless of dentin condition (≤ 4.5 MPa). Notably, the carious-IDS group demonstrated intermediate performance (6.10 ± 2.91 MPa), significantly higher than the DDS groups but inferior to sound dentin conditions (*p* < 0.01; Cohen’s d = 1.19, large effect size), highlighting a moderate clinical benefit of immediate sealing on carious dentin (Table 3).

After 6 months of water storage, SBS values decreased in all groups. The two-way ANOVA showed significant effects for both dentin condition and sealing strategy (*p* < 0.001). The sound-IDS group maintained the highest SBS values (12.22 ± 1.48 MPa; 95% CI: 10.91–13.53), indicating sustained adhesion. In contrast, the carious-IDS group dropped substantially (1.35 ± 0.22 MPa; 95% CI: 1.16–1.54), suggesting limited long-term effectiveness of IDS in demineralized substrates (Cohen’s d = 5.46 when compared to sound-IDS; very large effect).

Interestingly, the carious-DDS group (3.27 ± 2.46 MPa; 95% CI: 1.68–4.86) outperformed the carious-IDS group (*p* < 0.05), indicating that DDS may offer greater long-term stability when bonding to compromised dentin (Cohen’s d = 1.02; large effect). The sound-DDS and sound-control groups exhibited moderate bond strength values (5.49 ± 0.32 MPa and 5.42 ± 0.12 MPa, respectively), with no statistically significant difference between them (*p* > 0.05; Cohen’s d = 0.26, small effect). (Table 4).

**Figure 3:**
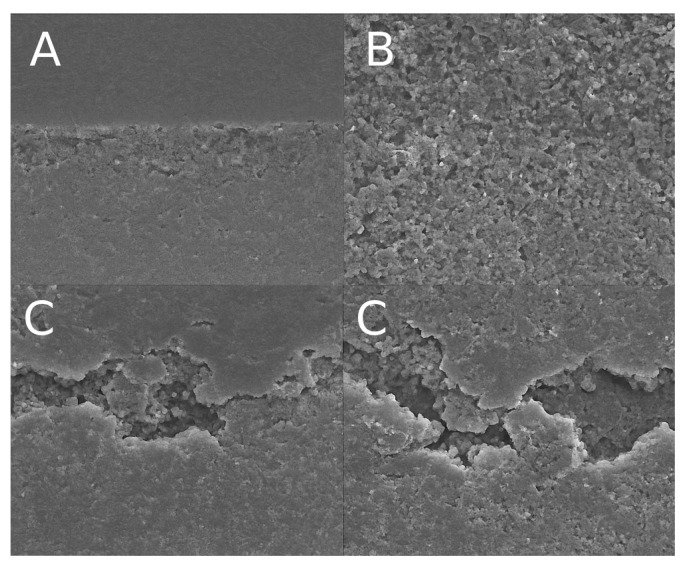
Representative SEM images of failure modes: (**A**) adhesive failure at the dentin–resin interface; (**B**) cohesive failure within dentin; and (**C**) mixed failure involving both the substrate and resin.

The failure modes of all specimens were assessed under a stereomicroscope at 40× magnification and classified as adhesive, cohesive (in dentin or resin), or mixed. Representative scanning electron microscopy (SEM) images of each failure mode are shown in Figure 3.

In groups bonded to sound dentin, a predominance of mixed failures was observed, particularly in the IDS groups. For example, in the sound-IDS group, 70% of the failures were mixed, 20% adhesive, and 10% cohesive. Similarly, the sound-control group showed 65% mixed failures and 35% adhesive failures. In contrast, specimens bonded to carious dentin exhibited a higher incidence of adhesive failures, especially after 6 months of aging. The carious-IDS group showed 85% adhesive failures, 10% mixed, and 5% cohesive, indicating a weaker hybrid layer and reduced interfacial integrity. The carious-DDS group also demonstrated a high proportion of adhesive failures (75%), supporting the reduced durability of bonding in demineralized substrates.

These trends suggest that immediate dentin sealing promotes more stable hybrid layer formation in sound dentin, while its effectiveness is limited in carious dentin, particularly under long-term aging conditions.

## 4. Discussion

The present study evaluated the shear bond strength (SBS) of indirect resin composite restorations bonded to sound and demineralized dentin using IDS and DDS techniques, both at 24 h and after 6 months of aging. The results confirmed that the IDS technique provided significantly higher bond strength than DDS when applied to sound dentin, while DDS showed better long-term performance in demineralized dentin.

These findings reaffirm the advantages of IDS in ideal substrates, as the technique allows for the formation of a stable adhesive interface before exposure to functional stress or contamination. Immediate sealing enables for the complete polymerization of the adhesive layer, which reduces the potential for hydrolytic degradation and minimizes interference from provisional cements, thereby enhancing the integrity of the dentin–resin bond [2,6,7].

IDS has been consistently shown to enhance the quality and durability of the resin–dentin bond [17]. Magne et al. (2005) demonstrated that IDS improves bond strength by eliminating early exposure to contaminants and allowing adhesive systems to polymerize under optimal conditions [5]. These findings are further supported by Falkensammer et al. (2014), who confirmed that IDS maintains adhesive strength even after thermocycling, simulating long-term clinical performance [18]. One of the notable clinical advantages of IDS is the significant reduction in postoperative sensitivity, a common consequence of fluid movement in exposed dentin tubules. By sealing the dentin immediately after preparation, IDS effectively minimizes dentinal fluid dynamics, thereby reducing the incidence of hypersensitivity. Hu and Zhu (2010) observed that patients treated with IDS reported substantially less sensitivity following cementation compared to those managed with DDS techniques [19].

Demineralized dentin, however, represents a major clinical challenge. The partially degraded collagen matrix, increased permeability, and morphological irregularities limit adhesive infiltration and polymerization efficiency. This could explain the higher prevalence of adhesive failures observed in the carious groups, particularly after aging. The loss of bonding effectiveness over time may also be attributed to water sorption, the hydrolytic nature of some adhesive monomers, and enzymatic degradation at the interface [20,21,22,23,24].

Interestingly, the DDS technique demonstrated relatively better durability on carious dentin after six months. Although DDS tends to produce lower initial bond strength due to temporary cement contamination or a lack of immediate hybridization, it may allow for improved substrate stability by the time of final cementation. This delay might reduce hydrolytic stress on the adhesive interface when applied to structurally compromised dentin, providing a more resilient bond over time [1,20,21,22,23,24].

One explanation is substrate maturation: caries-affected dentin is porous and demineralized, and immediate bonding may result in inadequate resin infiltration and increased water permeation [25]. However, by deferring adhesive application, the substrate may undergo partial remineralization and collagen fiber reorganization, enhancing mechanical properties and resin penetration later [19]. This parallels the stepwise caries removal approach, where interim stabilization improves dentin quality for the final restoration.

Failure mode analysis supported the mechanical findings. Mixed failures predominated in sound dentin, especially with IDS, indicating strong and balanced adhesion. In contrast, adhesive failures were most frequent in aged carious groups, particularly with IDS, highlighting the vulnerability of this technique under suboptimal substrate conditions. These observations confirm that the success of IDS is highly dependent on substrate quality and stability over time.

From a clinical perspective, these results suggest that adhesive strategies should be adapted to the condition of the remaining dentin. IDS remains the preferred approach for indirect restorations when sound dentin is available, as it offers higher bond strength and longevity. However, in cases involving demineralized dentin—such as after selective caries removal—DDS may offer greater long-term stability, especially when supported by strict moisture control and careful cleaning protocols.

Despite the controlled conditions of this in vitro study, several limitations must be acknowledged. First, only one adhesive and cement system were tested, which may limit the applicability of the findings across the wide variety of products used clinically. Aging was simulated exclusively through water storage without incorporating thermocycling or pH cycling, thus not fully replicating the thermal and chemical stresses experienced intraorally, which can impact the long-term durability of the adhesive interface. The use of flat dentin specimens, rather than anatomically relevant cavity preparations, may not accurately represent the complex dentin morphology and substrate heterogeneity encountered in clinical situations. Moreover, the study did not evaluate the potential influence of provisional materials or their removal on the dentin surface, which could affect bond strength in practice. Factors such as operator variability and technique sensitivity, known to significantly affect bonding outcomes, were not simulated or examined. Finally, the in vitro setting cannot fully replicate the dynamic oral environment, including factors like intraoral humidity, occlusal forces, and patient-specific biological variations, all of which may influence adhesive performance. Nevertheless, efforts were made to standardize procedures and minimize confounding variables to ensure methodological rigor.

Future research should explore the role of different adhesive systems, dentin pretreatments, and aging protocols to better understand the performance of IDS and DDS in compromised substrates. In vivo studies and randomized controlled trials are also essential to confirm these findings and to assess long-term clinical outcomes under functional load. To enhance the external validity and clinical relevance of findings, future studies should incorporate thermocycling and pH cycling protocols to better simulate oral environmental conditions. Additionally, evaluating a broader range of adhesive and cement systems would provide more comprehensive insights into their performance on demineralized dentin over time. Potential modifications to the IDS protocol could help overcome the bonding challenges associated with demineralized dentin. For example, pretreating the dentin surface with collagen cross-linking agents or enzymatic inhibitors may stabilize the exposed collagen matrix and reduce its degradation, enhancing adhesive infiltration and durability. Additionally, modifying adhesive formulations to include hydrophilic monomers or nanofillers could improve penetration into the porous dentin substrate. Applying multiple layers of adhesive or extending application time may also strengthen the hybrid layer in compromised dentin. Surface treatments such as selective etching or air abrasion prior to IDS could further increase micromechanical retention [26,27]. Future research should focus on testing these protocol adaptations under conditions simulating the oral environment, including thermal cycling and mechanical loading, to assess their potential in improving the long-term success of IDS on demineralized dentin.

## 5. Conclusions

Within the limitations of this in vitro study, it can be concluded that:

- ⁠IDS significantly enhances bond strength in sound dentin, outperforming DDS at both 24 h and after 6 months.
- ⁠⁠IDS ensures a stable and durable adhesive interface under optimal conditions.
- In demineralized dentin, IDS performance declines over time, with more adhesive failures after aging.
- DDS showed better long-term stability in carious dentin compared to IDS.
- An adhesive strategy should be adapted based on dentin condition:
  - The use IDS for sound dentin.
  - DDS is preferred for compromised or demineralized dentin.
- ⁠Clinical protocols must account for substrate differences to optimize restoration longevity.

## Figures and Tables

**Table 1:** Materials used for bonding and restorative procedures, including manufacturers and locations.

Material	Manufacturer	City, Country
Single Bond Universal adhesive	3M ESPE	St. Paul, MN, USA
Filtek Z250 XT resin composite	3M ESPE	St. Paul, MN, USA
Systemp Onlay provisional material	Ivoclar Vivadent	Schaan, Liechtenstein
Theracem dual-cure resin cement	Bisco	Schaumburg, IL, USA

**Table 2:** Three-way ANOVA results for shear bond strength (SBS, in MPa), evaluating the effects of dentin condition, aging, and sealing strategy.

Source of Variation	DF	H.H	MS	F	*p*
Dentin	1	172.337	172.337	50.957	<0.001
Aging	1	218.149	218.149	64.502	<0.001
Group	2	305.773	152.887	45.205	<0.001
Dentin × Aging	1	14.239	14.239	4.210	0.046
Dentin × Group	2	258.058	129.029	38.151	<0.001
Aging × Group	2	185.322	92.661	27.398	<0.001
Dentin × Aging × Group	2	7.434	3.717	1.099	0.341
Residual	32	595.506	18.610		
Total	39	926.375	23.753		

DF: degrees of freedom; H.H: sum of squares; MS: mean square (SS divided by DF); F-value (test statistic from ANOVA); and *p*-value (statistical significance).

**Table 3:** Shear bond strength (SBS) values (MPa) for each group at 24 h (mean ± standard deviation).

Group	Healthy Dentin (MPa)	Carious Dentin (MPa)
Control	A 13.85 (2.35) ^a^	
Immediate dentin sealing	A 13.69 (1.48) ^a^	B 6.10 (2.91) ^b^
Late dentin sealing	A 4.11 (1.68) ^b^	A 4.45 (2.76) ^b^

Different capital letters indicate statistically significant differences for column comparisons (*p* < 0.05). Different superscript lowercase letters indicate statistically significant differences for row comparisons (*p* < 0.05).

**Table 4:** Shear bond strength (SBS) values (MPa) for each group at 6 months (mean ± standard deviation).

Group	Healthy Dentin (MPa)	Carious Dentin (MPa)
Control	A 5.42 (0.12) ^b^	
Immediate dentin sealing	A 12.22 (1.48)	B 1.35 (0.22) ^b^
Delayed dentin sealing	A 5.49 (0.32) ^b^	A 3.27 (2.46) ^c^

Different capital letters indicate statistically significant differences for column comparisons (*p* < 0.05). Different superscript lowercase letters indicate statistically significant differences for row comparisons (*p* < 0.05).

## Data Availability

The original contributions presented in this study are included in the article. Further inquiries can be directed to the corresponding author.
